# The Effect of Public Healthcare Expenditure on the Reduction in Mortality Rates Caused by Unhealthy Habits among the Population

**DOI:** 10.3390/healthcare10112253

**Published:** 2022-11-10

**Authors:** José Manuel Santos-Jaén, Ana León-Gómez, María del Carmen Valls Martínez, Fernando Gimeno-Arias

**Affiliations:** 1 Department of Accounting and Finance, University of Murcia, 30100 Murcia, Spain; 2 Department of Finance and Accounting, University of Malaga, 29071 Málaga, Spain; 3 Mediterranean Research Center on Economics and Sustainable Development, 04120 Almería, Spain; 4 Economics and Business Department, University of Almeria, 04120 Almeria, Spain; 5 Department of Management and Finance, University of Murcia, 30100 Murcia, Spain

**Keywords:** healthcare spending, health resources, unhealthy habits, mortality, partial least squares structural equation modeling (PLS-SEM)

## Abstract

The health systems of developed countries aim to reduce the mortality rates of their populations. To this end, they must fight against the unhealthy habits of citizens, such as smoking, excessive alcohol consumption, and sedentarism, since these result in a large number of deaths each year. Our research aims to analyze whether an increase in health resources influences the number of deaths caused by the unhealthy habits of the population. To achieve this objective, a sample containing key indicators of the Spanish health system was analyzed using the partial least squares structural equation modeling (PLS-SEM) method. The results show how increasing public health spending and, thus, the resources allocated to healthcare can curb the adverse effects of the population’s unhealthy habits. These results have important implications for theory and practice, demonstrating the need for adequate investment in the healthcare system to reduce mortality among the population.

## 1. Introduction

In recent years, health has been perceived as one of the key tools in a country’s development process [1,2]. Therefore, one of the key policies for improving the development of a given country is to maintain, expand, and improve its health system [2,3]. Consequently, the main objective of a national health service is to achieve a healthier population by improving the quality of life and the health of its citizens [4], resulting in an increase in the life expectancy of its citizens [5]. In this context, research on health risk factors is a constant concern within the healthcare field, as an effective health service contributes to reducing mortality rates [6].

**Figure 1:**
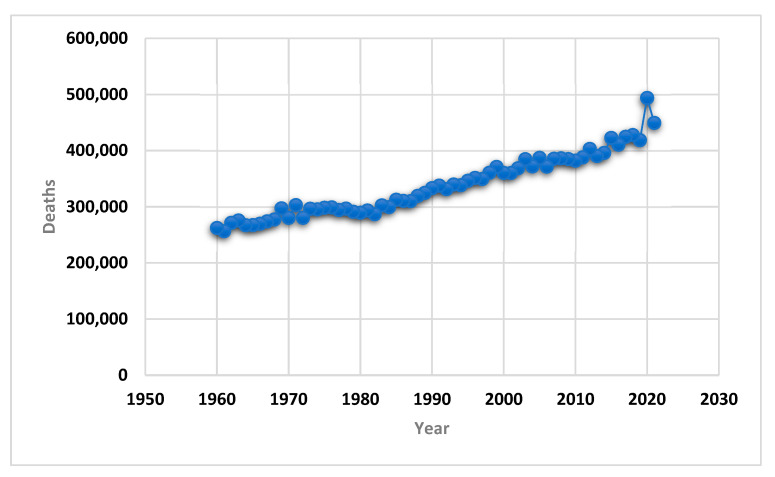
Evolution of the number of deaths in Spain from 1960 to 2021. Source: *Expansión* and the authors’ creation.

According to the newspaper *Expansión* [7], an average of 1231 deaths occurred daily in Spain in the year 2021. Figure 1 shows the upward trend in the number of deaths in Spain from 1960 to the present day. Specifically, 449,270 people died in 2021, 44,506 fewer than in the previous year, with the mortality rate falling from 10.40‰ to 9.49‰. The increase in mortality in 2020 was due to the COVID-19 pandemic, which caused an unexpected increase in the number of deaths [8]. However, if we look at the long-term evolution of the number of deaths in Spain, we see that there has been a prolonged upward trend since 1960, when it stood at 262,260 deaths. In recent years, life expectancy in Spain has increased notably [9], with an average in 2021 of 83.06 years (85.83 for women and 80.24 for men). These figures represent an increase of around 20% for both genders with respect to life expectancy in Spain in 1960. In relation to the causes of mortality, according to data from the European Health Survey [10] for the year 2020, around 50,000 people died in Spain from smoking-related diseases, some 20,000 died from excessive alcohol consumption, and almost 55,000 died as a result of physical inactivity.

**Figure 2:**
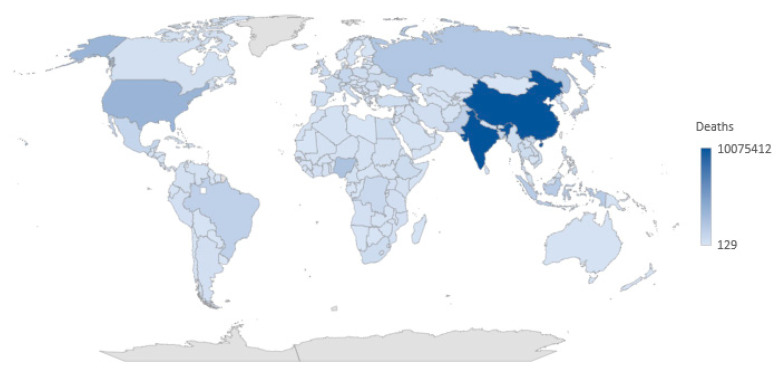
Global comparison of the number of deaths by country. Source: *Expansión* [7] and the authors’ creation.

In addition, Figure 2 shows the global comparison of the number of deaths by country. The intensity of the color represents the percentage of the number of deaths, with the countries shown in dark blue having the highest number of deaths, such as India (with a total of 10,075,412 deaths) and China (9,983,688 deaths). In contrast, the countries shown in light blue have the lowest number of deaths worldwide, such as Luxembourg (4489 deaths) and Iceland (2333 deaths). Spain has climbed up the world ranking of the number of deaths, moving from 159th in 2020 to 148th in the ranking in 2021, indicating that it has a high number of deaths compared to the other countries.

For the aforementioned reasons, many researchers have shown great interest in recent decades in examining which factors reduce mortality rates. Individual healthy lifestyle factors, such as not smoking, maintaining a healthy weight, moderating alcohol intake, engaging in regular exercise, and eating a healthy diet all reduce the risk of mortality [11,12,13]. Smoking has been identified as the second leading risk factor for death from all causes worldwide [14]. For this reason, many researchers have shown interest in this subject [15,16,17,18,19]. As a result of their research, it is estimated that up to half of all smokers worldwide die from smoking-related diseases [20], mainly lung cancer [15,21,22]. Similarly, physical inactivity and excessive alcohol consumption also increase mortality rates [23,24]. Sedentarism has also been established as a major health risk factor [25,26,27,28], affecting endothelial function, autonomic function, nitric oxide bioavailability, and progenitor cell mobilization [29,30,31] and leading to heart failure. Consequently, authors such as Wen et al. (2011) [32] and Belvederi et al. (2020) [33] promote exercise as a key tool for reducing mortality, as it reduces underlying diseases such as cachexia and musculoskeletal vascular problems [34]. Excessive alcohol consumption is also associated with a wide range of chronic and acute negative health outcomes, such as serious illnesses and traffic accidents, leading to an increased risk of mortality [35,36,37]. Therefore, previous studies such as those by White et al. (2020) [38] and Probst et al. (2020) [39] support the need to reduce the number of alcohol-related deaths. Along this line, an essential part of the measures for reducing mortality is based on carrying out good preventive measures. As stated by De Marinis et al. [40], the bulk of preventive medicine and health promotion in Spain is integrated with primary healthcare and is carried out by family doctors and nurses as part of their normal activity under the responsibility of regional health authorities.

The data from the Ministry of Health’s accounts system [41] show that, in recent years, spending on preventive healthcare has increased within the total healthcare expenditure in Spain, rising from 4.2% (EUR 1464 million) in 2003 to 5.4% (EUR 3811 million) in 2020. The preventive expenditure for 2020 is distributed as follows: 13% for maternal and child health, family planning services, and consultations; 4% for school health services; 25% for the prevention of non-communicable diseases; 1% for occupational medicine; and 57% for other public health services. Despite the increase in recent years, per capita spending on preventive healthcare in Spain is approximately half that of the EU27, with an average of EUR 53 for Spain versus EUR 102 for the EU27 [42]. Regarding spending at the level of the autonomous communities, the political priority of preventive and public healthcare differs greatly between the different communities and seems to be independent of income levels and total healthcare spending. The highest-level communities spend four or five times more per capita in terms of GDP and in relation to the total current healthcare expenditure. This clearly manifests the lack of cohesion and coordination within the Spanish national health system [43].

Regarding the measures adopted in recent years for reducing unhealthy habits among the population, preventive strategies and best practices have been implemented both nationally and within the different autonomous communities [44]. The following measures can be highlighted:

- Act 28/2005 and Act 42/2010 on the production, sale, and consumption of tobacco.
- The National Strategy for Nutrition, Physical Activity, and Prevention of Obesity (NAOS).
- The National Integral Plan for Physical Activity and Sports.
- The Comprehensive Tobacco Action Plan for Andalusia (PITA).
- The Strategy for Promoting Healthy Eating and Physical Activity (PASEAR), 2013–2018, in Aragon.
- The Physical Activity, Sports, and Health Plan (PAFES) in Catalonia.

These preventive actions have resulted in a lower number of deaths in Spain due to preventable and treatable causes compared to the EU average, mainly as a result of effective public health and prevention policies prior to the COVID-19 pandemic [42].

Moreover, poverty is a critical problem associated with deteriorating health and increased mortality [45]. Adverse economic conditions lead to lower household incomes [46] and fewer opportunities for engaging in healthy behaviors [47]. As a result, people living in poverty have limited access to healthcare [48]. Consequently, poverty, ill health, and mortality combine to form a vicious cycle that harms a substantial proportion of the population [49].

**Figure 3:**
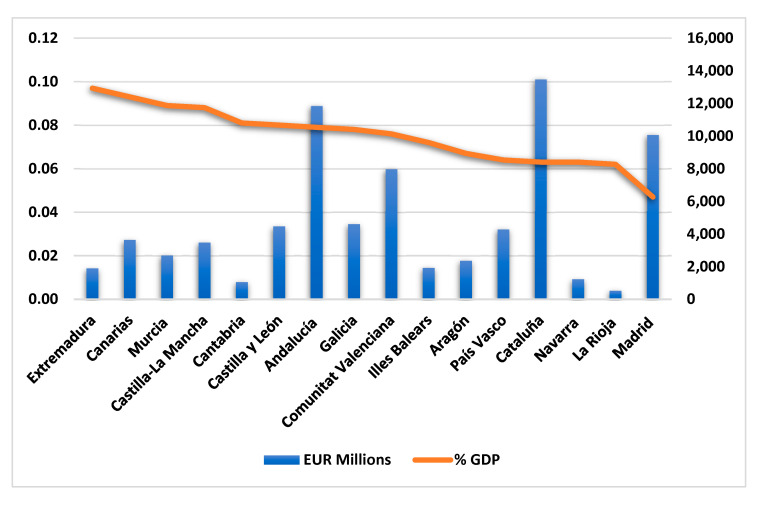
Public healthcare expenditure of the Spanish autonomous communities in 2020. Source: Ministry of Health.

On the other hand, one of the most significant current debates in the field of health policy is concerned with reducing this high mortality rate. Recent developments in this field have stimulated the need to increase public spending on health [50,51], as increasing health spending lowers the total mortality rate [52,53]. Despite having the fifth-largest healthcare budget within the European Union, Spain spends only 6.5% (as a proportion of GDP) on health policies [54]. It should also be noted that, in Spain, according to Law 14/1986, the autonomous communities are responsible for organizing their own healthcare systems [55], allocating varying amounts of financial and human resources [56]. Figure 3 presents the public healthcare expenditure of the autonomous communities for the fiscal year 2020. As can be seen, 45.8 percent of the sector’s public healthcare expenditure comes from three autonomous communities: Cataluña, Andalucía, and the Comunidad de Madrid, with EUR 13,473, EUR 11,858, and EUR 10,077 million, respectively. La Rioja, Cantabria, and the Comunidad Foral de Navarra have the lowest expenditure in absolute values. In contrast, the Comunidad de Madrid (with 4.7%), La Rioja (with 6.2%), Cataluña, and the Comunidad Foral de Navarra (both with 6.3%) have the lowest percentage of healthcare expenditure in relation to GDP. Thus, we can conclude that, despite the high investments in healthcare made by the Spanish autonomous communities, their share of GDP is relatively low.

As a consequence, although Spain has invested a lot of public money in healthcare, it is still insufficient to cover the needs of this sector [56,57], so more policies for increasing public spending on health are still required [58]. Thus, increasing public spending allows public hospitals to have greater flexibility when hiring additional staff [59] and to invest more in their infrastructures [60]. In this way, public health spending promotes an efficient allocation of resources that improves the use of human and material resources [61,62,63]. However, no clear evidence has been established as to how public health spending can counteract the adverse effects of the population’s unhealthy habits.

The main objective of this article is to examine whether an increase in public health spending favors a reduction in mortality caused by the population’s unhealthy habits by increasing resources for personnel, material resources, and infrastructures. To this end, the following research questions are posed: (1) Do unhealthy habits influence mortality in the population? (2) Does public health spending increase resources and thus reduce mortality? (3) Does public health spending reduce the adverse consequences of citizens’ unhealthy habits by reducing mortality?

To answer these questions, we developed a structural equation model based on the partial least squares (PLS-SEM) method to test our hypotheses on a sample of the 17 autonomous communities in Spain, with 136 observations. This paper has a double objective: confirmatory and predictive.

This article makes an essential contribution to the literature by considering public health spending as a key tool for reducing the mortality rate in Spain caused by unhealthy habits among the population. This is achieved by analyzing the relationship between public expenditure and mortality and incorporating the moderating effect of investment in personnel resources, material means, and infrastructure on the relationship between unhealthy habits and mortality. In addition, the results can help to raise awareness among policymakers of the need to make appropriate investments in the healthcare system, resulting in more available resources for hospitals and health centers, thereby reducing the number of deaths caused by the population’s unhealthy habits. This is the most important practical implication of this research, as well as the gap in the literature that it covers, which encompasses the need to provide empirical work to create solutions to the problem of the mortality rate of the Spanish population [64].

Following this introductory section, Section 2 shows the methodological aspects, Section 3 presents the results, Section 4 discusses these results, and, finally, Section 5 presents the main conclusions.

## 2. Methodology

### 2.1. Sample and Data Collection

Based on the ideas presented in the previous section, and to find out whether an increase in health resources reduces the mortalities caused by the population’s unhealthy habits, a PLS-SEM procedure was applied to check the following hypotheses:

**Hypothesis** **1 (H1).** *Unhealthy habits positively influence mortality.*

**Hypothesis** **2 (H2).** *Expenditure positively influences resources.*

**Hypothesis** **3 (H3).** *Resources negatively influence mortality.*

**Hypothesis** **4 (H4).** *Poverty positively influences mortality.*

**Hypothesis** **5 (H5).** *Resources moderate the relationship between unhealthy habits and mortality.*

**Figure 4:**
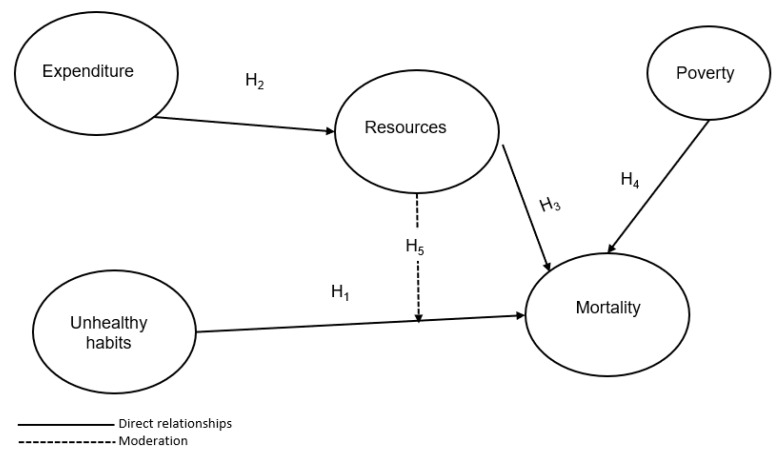
Conceptual model of the study.

From these hypotheses, we assembled the conceptual model shown in Figure 4.

In order to carry out this research, a database was constructed with information obtained from the National Health Key Indicators System (http://inclasns.msssi.es/; accessed on: 18 July 2022). These indicators, provided by the Spanish Ministry of Health, Consumption, and Social Welfare, are considered essential for determining the health of the population and the factors that influence it, as well as for analyzing how the public health system functions [65]. As a result, information was collected on the public healthcare expenditure, the resources available to the health system, and the population’s lifestyle, mortality rate, and poverty level. These data were obtained for the 17 autonomous communities that make up Spain for the years 2001, 2003, 2006, 2009, 2011, 2014, 2017, and 2020. These years were selected because they are the only ones for which data on the population’s lifestyle are available.

### 2.2. Variables

The variables used in this research are considered as Mode A (correlation weights) composite variables. This is because, based on how the data were obtained, a defining relationship was considered between the constructs and the indicators that form them [66]. In addition, the indicators that make up each construct are considered to be correlated [67]. Table 1 shows how each variable used in the proposed model was defined and composed.

Expenditure (public healthcare expenditure) is made up of a single indicator that shows the healthcare expenditure financed by the public system, whether it is produced by its own or external means through healthcare agreements. It is important to bear in mind that, as established by Santos-Jaén et al. [56], each autonomous community in Spain has complete authority over its own healthcare system. Therefore, healthcare spending is established in the general budgets of these autonomous communities, independently of one another.

Resources is made up of seven indicators that show the operational capacity of the healthcare system through the number of specialist doctors, operating rooms, and day hospital places, as well as the presence of several special types of equipment. As established by Valls Martínez et al. [55], resources in each autonomous community depend not only on current spending but also on past spending. A high-quality health service needs the availability of adequate resources to meet the population’s needs [4,68].

The unhealthy habits variable was constructed using three indicators that measure the population’s tobacco use, alcohol consumption, and sedentary lifestyles.

Considering that it is impossible to measure all possible causes of mortality, this variable consists of six indicators that measure mortality caused by six of the most frequent non-accidental causes of death among the population [69].

Finally, poverty was used as a control variable. This is made up of a single indicator that shows the poverty rate. A person is defined as being poor if his or her income does not reach the at-risk-of-poverty line.

### 2.3. Statistical Method

This analysis employed a partial least squares path modelling (PLS-PM) procedure [70]. The use of PLS-SEM in this research is appropriate because:

1. PLS-SEM does not require large samples or samples with a specific distribution [71];
2. PLS-SEM is particularly suitable for the study of moderations between variables [72];
3. PLS-SEM is recommended if secondary data are used [72];
4. PLS-SEM is a convenient tool for handling composite variable models [73].

PLS-SEM is an alternative to ordinary least square (OLS) regression, canonical correlation, or covariance-based structural equation modeling (SEM) of systems of independent and response variables [74]. PLS-SEM is a second-generation multivariate data analysis technique that gives a high level of confidence to research because of its statistical efficiency using robust and powerful software, such as SmartPls (used in this research) [75]. Its development has revolutionized empirical research, allowing many dependent relationships between independent and dependent variables to be examined simultaneously [76].

PLS-SEM estimates the relationships between constructs and determines how well the model explains the target variables. Its ability to estimate models with very complex relationships without high requirements has made it popular in many scientific areas (economics, social sciences, educational sciences, etc.) [77].

Two essential components characterize this technique: (1) the structural model and (2) the measurement model. The structural model is the guiding model showing the dependency relationships between independent and dependent variables. The measurement model shows the relationships between the constructs and their indicators [78].

Among the different structural equation models, PLS-SEM is based on the analysis of variance, which implies a more flexible modeling methodology, as it does not require rigorous parametric assumptions, mainly in the data distribution. Under PLS-SEM, confirmatory/exploratory and predictive models are established [79].

In the structural model analysis, to check the reliability of the items, their loadings have been studied (simple correlations of each indicator with its construct). The reliability of the constructs has been studied through the composite reliabilities (Cronbach’s alpha, composite reliability, and the Dijkstra–Henseler rho ratio). Cronbach’s alpha is an indicator of the reliability of a test based on its degree of internal consistency. Reliability, also called trustworthiness, is a property that refers to the absence of measurement errors, that is, to the degree of the consistency and stability of the data obtained in the measurement process with the same method. It indicates the degree to which the items of a test covary. It assumes that each indicator of a construct contributes similarly, as the loadings are set to unity. It measures the degree to which responses are consistent across questions of the same measure [80]. For its part, composite reliability allows the totality of constructs involved in the scale to be considered without assuming, like Cronbach’s alpha, that all constructs have the same weight. This index incorporates the loadings to create the value of each factor. To this end, it uses the loadings of each indicator in the same way as they are found in the causal model. That is, they are estimated as simple regressions of the effect variable by the ordinary least squares procedure [72]. Finally, the Dijkstra–Henseler rho ratio is an approximately exact measure of construct reliability, which usually lies between Cronbach’s alpha and the composite reliability. Hence, it may represent a good compromise if one assumes that the factor model is correct [72]. The convergent validity is studied through the average variance extracted (AVE). AVE measures the amount of variance that a construct can extract from its indicators in relation to the variance related to the measurement error [81]

Additionally, a blindfolding procedure (omission distance of 9) was used to check the general predictive relevance of the model. Blindfolding is a sample re-use technique, which systematically deletes data points and provides a prognosis of their original values. For this purpose, the procedure requires an omission distance D. A value for the omission distance D is between 5 and 12 [67]. The discriminant validity of the model is studied as follows: First, following the Fornell–Larcker criterion [81], it was verified that the correlations between each pair of latent variables did not exceed the square root of the AVE of each latent variable [82]. Secondly, the heterotrait–monotrait (HTMT) values were analyzed. HTMT represents the average of the correlations between indicators measuring the same construct relative to the average of the correlations of different constructs measuring different phenomena [72].

The analysis of the structural model will start with the study of the model’s goodness-of-fit through SRMR. SRMR represents the average of the correlations between indicators measuring the same construct relative to the average of the correlations of different constructs measuring different phenomena [72]. In order to rule out the existence of multicollinearity problems, the VIF will be used. VIF measures the degree of correlation between a variable and the rest of the variables in the model [72]. The coefficient of determination (R^2^) has been used to analyze the explanatory capacity of the model. R^2^ is the proportion of variation in a dependent variable that the statistical model predicts. Finally, f^2^ is used to measure the effect size [83]. f^2^ measures the degree to which an exogenous construct helps explain a given endogenous construct in terms of R^2^.

Data corresponding to the 17 autonomous communities in Spain for the years 2001, 2003, 2006, 2009, 2011, 2014, 2014, 2017, and 2020 were considered, with the final sample comprising 136 observations.

Although the sample size is not very large (n = 136), we checked the adequate statistical power of the investigation using the G*Power 3.1.9.2 software [84]. For this purpose, we performed an a priori analysis assuming a significance level of 5%, an effect size of 0.15, and a statistical power of 80%. The results indicate the need for a sample of at least 68 observations to validate the effects of the proposed model [83]. Therefore, the sample size used is satisfactory.

Finally, the SmartPLS 3.3.3 software [85] was used to test the hypotheses put forward in the model using a bootstrapping procedure with 10,000 bootstrap samples.

## 3. Results

According to Hair et al. [67] the analysis of this model is performed in three steps: analysis of the measurement model, analysis of the model structure, and analysis of the moderation.

### 3.1. Evaluation of the Measurement Models

The reliability of the indicators, the composite reliability, the convergent validity, and the discriminant validity were examined to test the reliability and validity of the constructs [86].

To check the reliability of the individual items, their loadings were analyzed, verifying that they all exceed the minimum value of 0.7 [87]. As can be seen in Table 2, this occurs in most cases. Moreover, for the cases where this does not occur, they at least exceed the value of 0.4. These values can be maintained if they are not detrimental to composite reliability [88], as in this model. The reliability of the constructs is satisfied since the composite reliabilities (Cronbach’s alpha, composite reliability, and the Dijkstra–Henseler rho ratio) are greater than 0.7 [67]. In addition, the convergent validity is satisfactory because the average variance extracted (AVE) measures are above 0.5 [89]. Additionally, a blindfolding procedure (omission distance of 9) was used to check the general predictive relevance of the model. As can be seen in Table 2, the values of Q2 are above 0, thereby confirming the predictive relevance of the model [90].

Next, two tests were performed to confirm the discriminant validity of the model (see Table 3). First, following the Fornell–Larcker criterion [81], it was verified that the correlations between each pair of latent variables did not exceed the square root of the AVE of each latent variable [82]. Secondly, the heterotrait–monotrait (HTMT) values were analyzed. These values vary between 0.088 and 0.820. Therefore, they do not exceed the maximum recommended value of 0.85 [91].

### 3.2. Evaluation of the Structural Models

In order to test the goodness-of-fit of the model, it was verified that the standardized root mean square residual (SRMR) does not exceed the value of 0.08 [92,93]. Moreover, the variance inflation factor (VIF) values were analyzed to discard the existence of collinearity problems between constructs. As can be seen in Table 4, these values range from 1 to 2.748. Therefore, they do not exceed the maximum recommended value of 3.0 [67].

Following the indications of Hair et al. [94], we continue the analysis of the structural model by studying the sign, magnitude, and significance of the paths, the coefficient of determination (R^2^), and the effect sizes (f^2^).

The results in Table 4 show that unhealthy habits has a significant positive effect on mortality (β = 0.618 ***), supporting H1. Likewise, expenditure has a significant positive effect on resources (β = 0.587 ***), supporting H2. On the other hand, resources has a significant negative effect on mortality (β = −0.268 ***), thereby confirming H3. Finally, the findings suggest that poverty, as a control variable, has a positive and significant effect on mortality (β = 0.083 **), supporting H4.

The coefficient of determination (R2) was used to analyze the explanatory capacity of the model. R^2^ displays, via the predicting variables of an endogenous construct, how these can explain the variance [72]. Values of 0.60, 0.33, and 0.19 are considered as substantial, moderate, and weak [83,95]. The results show that the model explains 34.50% of the variance in resources and 74.30% of the variance in mortality. Hence, the model’s explanatory power in the case of mortality is excellent.

Finally, f^2^ is used to measure the effect size [83]. f^2^ measures the degree to which an exogenous construct helps explain a given endogenous construct in terms of R^2^. A small effect is considered to exist if the value fluctuates between 0.02 and 0.15, which is the case for resources on mortality. A medium effect is considered to exist if the value fluctuates between 0.15 and 0.35. Finally, if the value is greater than 0.35, then the effects of unhealthy habits on mortality and those of expenditure on resources are considered to be large [83].

### 3.3. Moderating Effect

**Figure 5:**
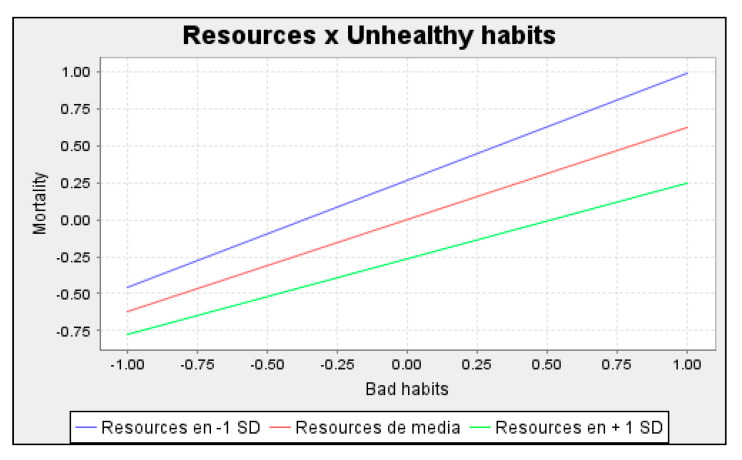
The moderating effect of resources on the relationship between unhealthy habits and mortality.

Figure 5 shows the interaction analysis results. To examine the conditional moderating effect of resources in the relationship between unhealthy habits and mortality, this study followed the procedure established by Ali et al. [96]. The findings support H5, such that the positive effect of resources on mortality is moderated by resources (β = 0.107 **). Figure 5 shows in detail the slope of the moderating effect. As can be seen, the effect of unhealthy habits on mortality is greater when fewer resources are available. As both a rule of thumb and an approximation, the slope for the relationship between unhealthy habits and mortality on the lowest level of resources is calculated as a simple effect (i.e., 0.618) minus the interaction effect (0.107). In contrast, the slope of the high level of resources is calculated as the simple effect (e.g., 0.618) plus the interaction effect (0.107). Hence, higher resources produced a weaker relationship between unhealthy habits and mortality.

**Figure 6:**
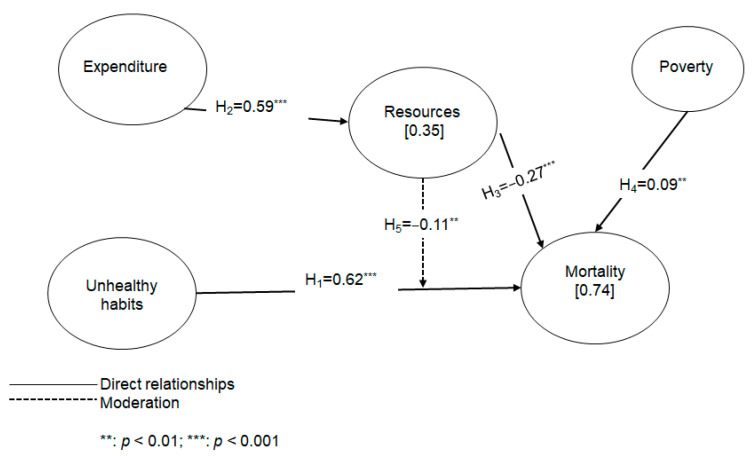
Results.

To summarize the results, Figure 6 presents the standardized path coefficients and R^2^.

## 4. Discussion

The main objective of this article is to examine whether an increase in public health spending favors a reduction in mortality caused by unhealthy habits among the population through a resulting increase in resources in terms of personnel, material means, and infrastructures. To do so, we developed a structural equation model based on the partial least squares (PLS-SEM) method to test our hypotheses on a sample of the 17 autonomous communities that make up Spain, with a total of 136 observations.

The results show how unhealthy habits among the population increase the risk of mortality. These results confirm those of previous studies that concluded that individual lifestyle factors, such as non-smoking, maintaining a healthy weight, moderate alcohol consumption, regular exercise, and a healthy diet, reduce the risk of mortality [5,6,7]. Similarly, it was also shown that the control variable poverty also increases the risk of mortality. Consequently, poverty, poor health, and mortality combine to form a vicious cycle that harms a substantial proportion of the population [49].

Moreover, this study yields results that underline the conclusions of much of the previous work in this field in considering public spending on healthcare as a key tool for reducing the mortality rate in Spain caused by the population’s unhealthy habits [62,63]. Public health spending favors investing in human and material resources in health centers [60]. This will improve the quality of healthcare services, thereby reducing the mortality rate [50,97].

Finally, our results suggest that a good health service can counteract the adverse effects of unhealthy habits, especially mortality. Therefore, not only is it necessary to invest in awareness campaigns to reduce these unhealthy habits, but it is also necessary to equip the health system with the means to counteract their impact on mortality.

## 5. Conclusions

This research makes an important contribution to the public health literature and research by incorporating the study of the moderating effect of investments in health resources on the relationship between a population’s unhealthy habits and mortality, thereby filling a gap in the existent literature.

The results may help to raise awareness among politicians of the fact that adequate investment in healthcare is needed. In relation to the problem of unhealthy habits among the population, this study shows that a good health service can counteract their adverse effects, especially mortality. Therefore, not only is it necessary to invest in awareness campaigns to reduce these unhealthy habits, but it is also necessary to provide the health system with the necessary means to counteract their impact on mortality.

This research has some limitations, which could be the subject of future lines of research, which are discussed below. First, the study was carried out only in Spain. In the future, this study could be replicated in other geographical areas, since the functioning of the health system and the population’s habits vary from country to country. Second, this study deals with the health system (expenditure and resources) as a whole. In the future, research dealing with the health system in a disaggregated manner (primary care, hospitals, specialized consultations, etc.) could be carried out. Third, bearing in mind that the aim of the research was to describe the impact of public health spending on mortality caused by unhealthy lifestyle habits, such as smoking, alcohol consumption, and sedentarism, future research could try to access and use data on primary, secondary, and tertiary prevention. In our case, this was not possible, as this information was not available. Likewise, it would be advisable for future research to use variables that measure policies implemented for limiting and preventing tobacco and alcohol consumption, since these policies require a public expenditure that is as important as that of hospital funding.

## Figures and Tables

**Table 1:** Measurement variables.

EXPENDITURE	
EXP_1	Health expenditure (public) per capita
RESOURCES	
RES_1	Specialist doctors per 1000 inhabitants
RES_2	Operating rooms per 1000 inhabitants
RES_3	Nuclear Magnetic Resonance Equipment per 1000 inhabitants
RES_4	CT equipment per 1000 inhabitants
RES_5	Hemodialysis equipment per 1000 inhabitants
RES_6	Hemodynamic equipment per 1000 inhabitants
RES_7	Day hospital places per 1000 inhabitants
UNHEALTHY HABITS	
BH_1	Tobacco (percentage of smokers)
BH_2	Alcohol (percentage of at-risk drinkers)
BH_3	Inactivity in leisure time (prevalence of sedentary behavior among the adult population)
MORTALITY	
MOR_1	Ischemic heart disease mortality rate
MOR_2	Cerebrovascular disease mortality rate
MOR_3	Cancer mortality rate
MOR_4	Chronic liver disease mortality rate
MOR_5	Chronic obstructive pulmonary disease mortality rate
MOR_6	Pneumonia and influenza mortality rate
POVERTY	
POV_1	Poverty rate

**Table 2:** Measurement model.

TOTAL	Mean	SD	Loading	*t*-Student *	Q^2^	α	ρA	ρC	AVE
EXPENDITURE									
EXP_01	1409.669	238.477	1.000	24.486					
RESOURCES					0.144	0.816	0.845	0.861	0.575
RES_1	1.806	0.296	0.702	14.582	0.298				
RES_2	8.952	1.299	0.645	8.793	0.062				
RES_3	0.996	0.416	0.854	40.140	0.192				
RES_4	1.556	0.307	0.801	21.929	0.235				
RES_5	9.713	3.752	0.567	7.787	0.050				
RES_6	0.458	0.169	0.604	9.565	0.029				
RES_7	0.305	0.183	0.601	9.119	0.143				
UNHEALTHY HABITS						0.702	0.734	0.742	0.510
BH_01	25.041	4.023	0.904	64.242					
BH_02	2.394	1.455	0.416	5.584					
BH_03	42.078	9.783	0.735	12.103					
MORTALITY					0.472	0.889	0.918	0.920	0.666
MOR_01	83.22	27.93	0.932	74.098	0.606				
MOR_02	74.828	27.619	0.906	66.703	0.723				
MOR_03	247.431	21.083	0.822	32.854	0.446				
MOR_04	10.558	3.309	0.806	20.891	0.309				
MOR_05	35.769	9.277	0.873	40.198	0.536				
MOR_06	21.369	6.243	0.465	6.163	0.214				
POVERTY									
POV_01	20.109	6.611	1.000	16.783					

Significance and standard deviations (SD) performed using a bootstrapping procedure with 10,000 bootstrap samples. Q2: cross-validated redundancies index performed by a nine-step distance-blindfolding procedure. α: Chronbach’s alpha; ρA: Dijkstra–Henseler’s composite reliability; ρC: Jöreskog’s composite reliability; AVE: Average Variance Extracted; * All loadings are significant at the 0.001 level.

**Table 3:** Discriminant validity.

		I	II	III	IV	V
I	EXPENDITURES	**1.000**	*0.605*	*0.715*	*0.622*	*0.088*
II	RESOURCES	0.587	**0.889**	*0.526*	*0.820*	*0.299*
III	UNHEALTHY HABITS	−0.542	−0.790	**0.814**	*0.634*	*0.288*
IV	MORTALITY	−0.588	−0.719	0.734	**0.816**	*0.264*
V	POVERTY	−0.088	−0.213	0.139	0.209	**1.000**

HTMT ratio over the diagonal (italics). Fornell–Lacker criterion: square root of AVE in diagonal (bold) and construct correlations below the diagonal.

**Table 4:** Path coefficients (b) and statistical significance.

TOTAL	Path	SD	T-Value	f^2^	95CI	VIF	H	Supported
Direct effects								
Unhealthy habits -> Mortality	0.618	0.074	8.370 ***	0.554	[0.487; −0.729]	2.682	H1	Yes
Expenditure -> Resources	0.587	0.053	11.153 ***	0.527	[0.488; −0.663]	1.000	H2	Yes
Resources -> Mortality	−0.268	0.070	3.808 ***	0.102	[−0.386; −0.158]	2.748	H3	Yes
Control variable paths								
Poverty -> Mortality	0.083	0.037	2.275 **	0.025	[0.025; 0.146]	1.075	H4	Yes
Indirect effects								
Moderating effects								
Resources x Unhealthy habits -> Mortality	−0.107	0.042	2.563 **	0.042	[−0.173; −0.036]	1.030	H5	Yes

R2 [95% CI in brackets]: Resources: 0.345 [0.238; 0.440]; Mortality: 0.743 [0.673;0.789]. Blindfolding Q2 index is shown in Table 1; Standardized path values reported; f2: size effect index; 95PCI: 95% percentile Confidence Interval; VIF: Inner model Variance Inflation Factors. Significance, *t*-Student, and 95% bias-corrected CIs were performed by a bootstrapping procedure with 10,000 repetitions; ** *p* < 0.01; *** *p* < 0.001.

## Data Availability

The data used in this research can be obtained from the Spanish Ministry of Health, Consumer Affairs, and Social Welfare website http://inclasns.msssi.es/, accessed on 17 July 2022.
